# Pelvic retroperitoneal pleomorphic hyalinizing angiectatic tumor (PHAT) of soft tissue: a case report

**DOI:** 10.1186/s12880-016-0130-3

**Published:** 2016-04-05

**Authors:** Zhi-gang Chu, Meng-qi Liu, Zhi-yu Zhu, Fa-jin Lv, Yu Ouyang

**Affiliations:** Department of Radiology, The First Affiliated Hospital of Chongqing Medical University, 1# Youyi street, Chongqing, 400016 China; Department of Stomatology, The First Affiliated Hospital of Chongqing Medical University, 1# Youyi street, Chongqing, 400016 China

**Keywords:** Pleomorphic hyalinizing angiectatic tumor, Computed tomography, Magnetic resonance imaging

## Abstract

**Background:**

Pleomorphic hyalinizing angiectatic tumor (PHAT) is a rare soft tissue tumor of low malignant potential, which most often arises in the lower extremities. Lesions occurred in other anatomic locations have been rarely reported. Moreover, their imaging features have not been well discussed. Here we report a case of PHAT arising primarily in the pelvic retroperitoneum. To our knowledge, this is the first radiological description for retroperitoneum PHAT.

**Case presentation:**

A 26-year-old female was referred to our hospital for evaluation of a pelvic mass incidentally noted in routine pre-pregnancy ultrasonography examination. Magnetic resonance imaging (MRI) and computed tomography (CT) scan revealed an irregular mass with clear boundary in the pelvic retroperitoneum. Its signal intensity or density was inhomogeneous. On MRI images, it mainly showed isointense and slight hypointense on T1 weighted image and isointense and hyperintense on T2 weighted image. On contrast-enhanced images, it showed marked but heterogenous enhancement. With the delay time increasing, the enhanced area in the lesion increased but the CT value decreased. Dilated vessels and hemorrhage were detected in the tumor. With patience and careful separation, it was completely excised with great amount of bleeding during operation. Pathological and immunohistochemistry analysis confirmed the diagnosis of PHAT of the soft parts. We found no evidence of recurrence 18 months after operation.

**Conclusion:**

We present an extremely rare case of PHAT arising primarily in the pelvic retroperitoneum. To our knowledge, this is the first radiological description for retroperitoneum PHAT. The provided information is useful for summarizing the characteristics of this kind of tumor. It should be included in the differential diagnosis of a well-defined, inhomogenously enhanced hypervascular soft-tissue mass in pelvic cavity.

## Background

Pleomorphic hyalinizing angiectatic tumor (PHAT) of soft parts is a rare tumor described first in 1996 by Smith et al. [1]. To date, different cases have been reported and which usually located at the lower extremities of adults. Other rarer sites involved were forearm, shoulder, axilla, buttock and buccal mucosa [2]. As this lesion in the present study occurred in an extremely rare location, it is necessary to further enrich the relevant information for diagnosing and managing. In this case, we introduce a large pathologically confirmed PHAT in pelvic retroperitoneum with focus on describing its imaging features. To our knowledge, this was the first reported case of PHAT which occurred in this site and had comprehensive radiological data.

## Case presentation

A 26-year-old female was referred to our hospital for evaluation of a pelvic mass incidentally noted in routine pre-pregnancy ultrasonography examination. The lesion did not cause any discomfort, and the patient did not report any symptom. She and her family members had not experienced such lesion in the past. General examination was unremarkable and laboratory findings were normal.

**Fig. 1:**
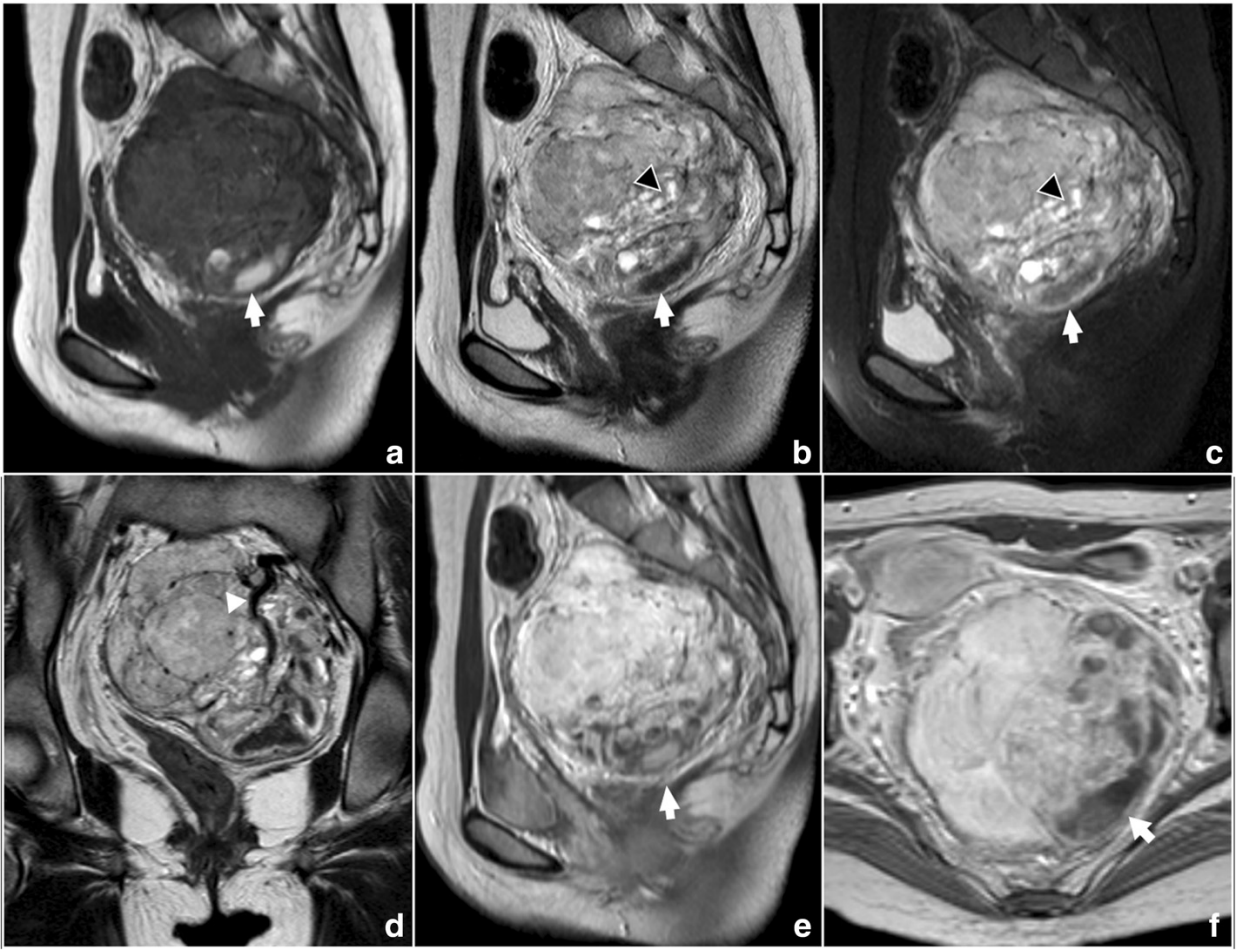
MRI examination of the pelvic cavity. Sagittal T1-weighted image (**a**) shows a well-defined lobulated mass with isointense and slight hypointense in the pelvic retroperitoneum. The lower part of the mass shows some areas with hyperintense (arrow). Sagittal T2-weighted image (**b**) and T2-weighted image with fat saturation (**c**) show the mass with inhomogeneous isointense and hyperintense. Cystic degeneration could be detected in the inner part (arrowhead). The lower part of the mass showed some areas with hypointense (arrow). Coronal T2-weighted image (**d**) shows large vessels with flow-void sign (arrowhead). Post-contrast T1-weighted images (**e**-**f**) show the mass with intense heterogeneous enhancement. The lower part with hyperintense on T1-weighted image and hypointense on T2-weighted image shows no significant enhancement (arrow)

Magnetic resonance imaging (MRI) of the pelvic cavity was performed. MRI examination revealed an irregular mass with clear boundary in the pelvic retroperitoneum (Fig. 1). It located in the front of sacrum and behind uterus and rectum. The oppression and displacement of neighbor organs were found. No direct invasion was detected. This mass measured 9.4 cm (anterior-posterior), 9.1 cm (transverse), and 9.5 cm (craniocaudal). The signal intensity of this lesion was inhomogeneous. It mainly showed isointense and slight hypointense on T1 weighted image and isointense and hyperintense on T2 weighted image. On contrast-enhanced images, it showed significant but heterogenous enhancement. The left and lower part of the mass showed some areas with hyperintense on T1 weighted image and hypointense on T2 weighted image. On contrast-enhanced images, it showed no enhancement. This abnormal signal indicated the presence of hemorrhage within the tumor. In addition, flow-void sign was identified at the lesion core and margin. The uterus, bilateral ovaries and rectum had no abnormalities.

**Fig. 2:**
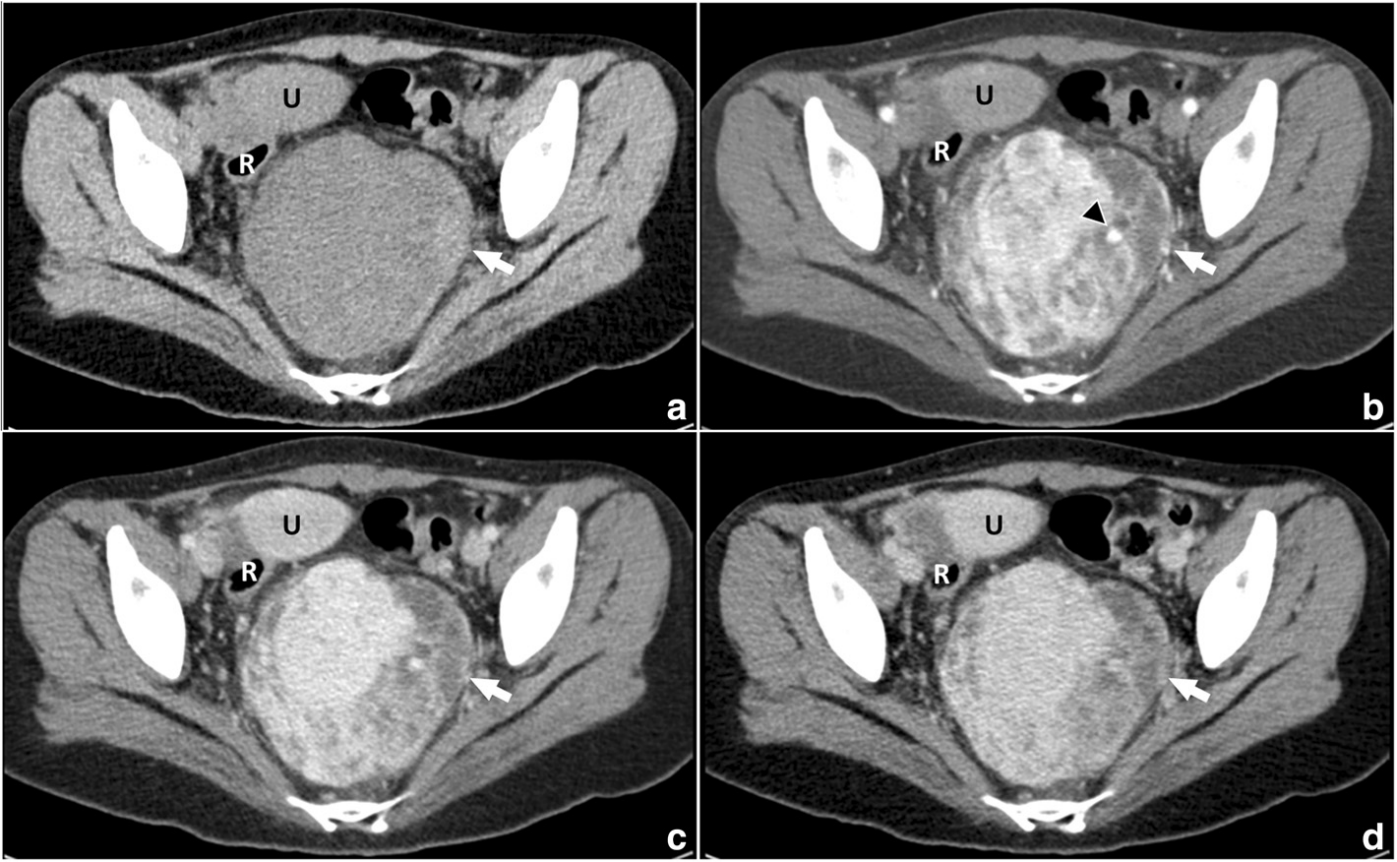
CT examination of the pelvic cavity. Plain CT scan (**a**) shows a well-defined mass with slightly inhomogenous density in the pelvic retroperitoneum. The left part of the mass has higher density (arrow). On contrast-enhanced CT images (**b**), it shows marked but heterogenous enhancement during arterial phase. Dilated feeding artery shows clearly in the mass (arrow). The enhanced area increases but the CT value decreases in the following phases (**c**-**d**). Areas with higher density showed on plain CT scan has no obvious enhancement (arrow). Some irregular area without obvious enhancement in the inner part is also detected. U: uterus, R: rectum

Subsequently, computed tomography (CT) examination was performed for further understanding this lesion and its surroundings, especially the adjacent vessels. Pre-contrast CT scan demonstrated a well-defined, irregular soft tissue mass in the same location showed on MRI (Fig. 2). The internal density was slightly inhomogenous, with CT values ranging from 34 to 69 HU. The left and lower parts of the mass demonstrated higher density with CT values ranging from 51 to 69 HU. No calcification was identified. On contrast-enhanced CT images, it showed marked but heterogenous enhancement during arterial phase, with a maximum CT value of 228 HU, which was slightly lower than that of the adjacent vessels (CT value: 284 HU). Dilated feeding arteries and drainage veins showed clearly in the mass core and margin. The enhanced area increased but the CT value decreased in the following phases. However, areas with higher density showed on plain CT scan had no obvious enhancement. This manifestation also indicated there was hemorrhage in the mass. In the peripheral parts around hemorrhage, some irregular areas without obvious enhancement were also detected. No other masses or abnormalities were found in abdomen. No enlarged lymph nodes were detected around the mass.

**Fig. 3:**
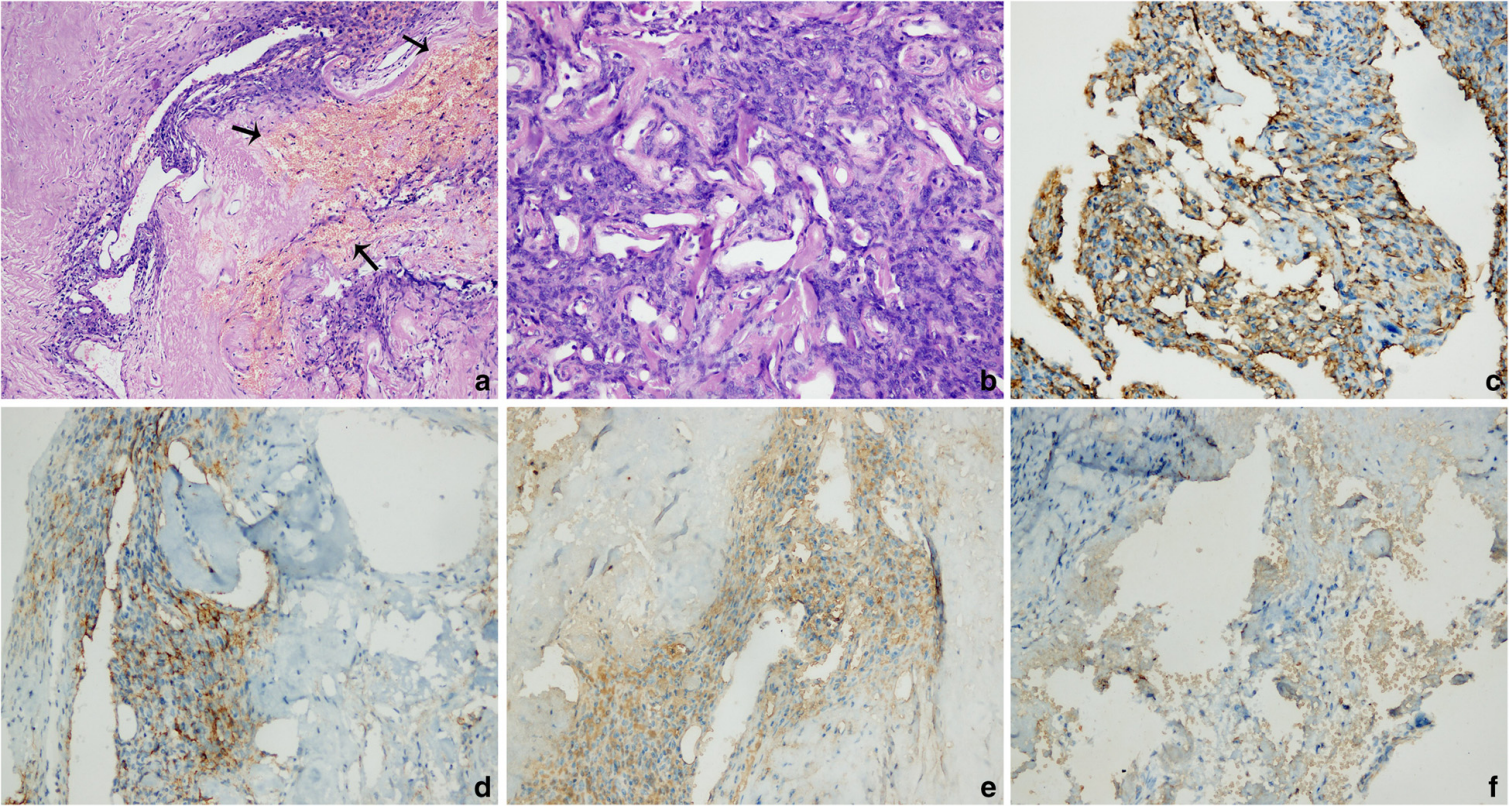
Hematoxylin and eosin stain (**a**-**b**) clusters of dilated thin-walled vessels. Pleomorphic tumor cells were distributed among these dilated vessels (A: original magnification, ×100, B: original magnification, ×200). Patchy hemorrhage is detected (arrows). Immunohistochemistry shows positive reactivity for CD34 (**c**), CD99 (**d**), CD117 (**e**) and vimentin (**f**) in the tumor cells (original magnification, ×200)

It could be confirmed this mass with abundant blood supply did not arise in the organs in pelvic cavity according to the imaging examinations. Then, the lesion was radically excised. During surgery, the mass was found behind the posterior pelvic peritoneum. It is very irregular and tightly adhered to the adjacent tissue. No invasion was detected. There were many vessels in the surface of tumor. With patience and careful separation, it was completely excised with great amount of bleeding during operation. The cut surface was grayish-white in color and necrosis and blood clots were seen in its inner part. It showed solid tissue with a somewhat crisp texture. The histopathology findings showed clusters of dilated thin-walled vessels prominent hyalinization of the vessel walls, and a perivascular and intercellular ground substance. Pleomorphic tumor cells were distributed among these dilated vessels. The mitotic counts was 3/10 HPF. Hemorrhage was also detected. Immunohistochemistry revealed that the tumor cells were positive for vimentin, cluster of differentiation (CD) CD34, CD99, CD117 (focal), and B‑cell lymphoma 2 (BCL-2), whereas S-100 protein, CD56, smooth muscle actin (SMA), Desmin, DOG-1, ALK-1 and actin was negative (Fig. 3). The Ki-67 labeling index was 5 %. The final diagnosis was pleomorphic hyalinizing angiectatic tumor (PHAT) of the soft parts.

The postoperative period was uneventful. In the 18 months since the operation, the patient has continued to have no occurrence.

## Discussion

Among the reported cases of PHAT in the English language literatures, the patients’ sex and age had no significant tendency [3]. A majority of tumors were located in the subcutaneous tissue, mainly in extremities [3–6]. Patients usually had no significant symptoms except showing a local, slowly growing mass. The disease time course and size of tumors varied significantly, which were closely related to their locations [4]. After operation, the local recurrence was relatively common [3, 4], but distal metastasis had not been described. In this study, the mass located in pelvic retroperitoneal space, which is a deep location consisted of loosened tissue. In addition, it did not cause any obvious symptom. Thus, this lesion was very large when it was accidentally discovered.

The CT and MRI manifestations of PHAT arising in neck and extremities have been reported previously [6–8]. However, radiological features of this kind of tumor were still not well understood. It is necessary to enrich the relevant knowledge for its diagnosis and management. We found the image findings of this case reported here had some features. Plain CT and MRI scan showed it was a well-defined mass with inhomogenous dense or signal. Previously reported cases occurred in the neck or renal hilum also had clear boundary [7, 9]. However, some cases arising in extremities were ill-defined [6]. On contrast-enhanced images, this tumor markedly but heterogenously enhanced and many dilated vessels could be detected in it. These manifestations were also illustrated in the previous reported cases [6, 7], which could be seen as the common features of PHAT. Regarding the mass with dilated vessels, preoperative embolization is helpful for reducing intraoperative bleeding and increasing cut rate and safety of operation. In addition, hemorrhage in this mass showed on CT and MRI was confirmed by operation and pathological examination. This finding has been reported in previous cases. We suspect it is related to the abundant blood supply and size of the lesion [5, 10]. The larger one might be more prone to have hemorrhage. Beside areas with necrosis and hemorrhage, some irregular areas without obvious enhancement were also detected. Pathologically, it was confirmed to be the prominent hyalinization area of vessel wall and the perivascular ground substance. In summary, the imaging features could well reflect its pathological nature.

Previously reported shapes of masses were usually lobulated, round or oval [3, 4, 11–13]. This large tumor reported here was lobulated. Generally, most of the PHATs were non-encapsulated and incorporated the surrounding normal tissue into the tumor tissue [3, 6]. A few cases had a thin and complete capsule, which manifested smooth and clear boundaries. Though the present case had smooth boundaries, no capsule was detected in operation. In contrast, it tightly adhered to the adjacent tissue. The possible reason was that retroperitoneal fat around the lesion made it showing distinct borders. However, fat space was deficiency among the muscles in the extremities. This tumor has potential for local recurrence. Thus, wide local excision and close follow up are the best approaches for management.

PHAT requires a differential diagnosis with other vascular or hypervascular masses, which includes solitary fibrous tumor (SFT), paraganglioma and gastrointestinal stromal tumor (GIST). SFTs in pelvic cavity appear as well-defined masses with intense heterogeneous enhancement that persists on delayed phase images. Necrosis, hemorrhage, or cystic change may be seen [14]. Paraganglioma appears as a well-defined hypervascular mass in the pelvic retroperitoneum along the course of the common iliac vessels. These tumors may be heterogeneous with foci of calcification. At MR imaging, it appears as areas of low T1 and high T2 signal intensity and shows significant enhancement [15]. Primary pelvic retroperitoneal GIST is extremely rare. They appear as large, hypervascular enhancing masses that frequently harbor areas of necrosis, hemorrhage, or cystic degeneration [16]. The signal of paraganglioma is significantly different from that of PHAT. PHAT usually has intense arterial enhancement and its degree of enhancement is higher than that of SFT and GIST. In addition, hypervascular alveolar soft part sarcoma (ASPS) should also be considered as a differential diagnosis. On contrast-enhanced images, ASPS and PHAT have similar manifestations, while the former usually shows high signal on T1 and T2 weighted images [17–19].

Immunohistochemically, the PHAT is positive for CD34, vimentin, CD99, and VEGF but negative for S-100 protein [1]. The present study showed the similar results. However, these findings are not specific for its diagnosis. Positive findings of CD34 and vimentin only indicate a mesenchymal tumor. The negative S-100 protein excludes a schwannoma from the differential diagnosis, which is the most common lesion that can be confused with this entity [9]. SFT shows a similar immunoprofile to PHAT, both express vimentin, CD34, and CD99 [20]. However, the histopathological findings of PHAT are obviously different from those of SFT [21]. Malignant fibrous histiocytomas can occasionally be CD34 positive but are negative for CD99 [9]. In addition, the negative CD56, SMA, Desmin, DOG-1, ALK-1 and actin in PHAT is distinguishable from neurogenic or myogenic tumors and GIST on pathology.

## Conclusion

In conclusion, to the best of our knowledge, this is the first radiological description for retroperitoneum PHAT. This newly recognized, rare neoplasm has some imaging features corresponding to its pathological nature. Although its locations and imaging features are variable, the provided information is useful for summarizing the characteristics of this kind of tumor. It should be included in the differential diagnosis of a well-defined, inhomogeneously enhanced hypervascular soft-tissue mass in the pelvic cavity.

### Consent

We pledged to abide by the declaration of Helsinki (2000 EDITION) in accordance with the relevant medical research rules of China in the study. IRB at The Affiliated Hospital of Chongqing Medical University approved this study.

### Availability of data and materials

Data are available on request from the corresponding author.

## Abbreviations

PHAT: pleomorphic hyalinizing angiectatic tumor
MRI: magnetic resonance imaging
CT: computed tomography
SFT: solitary fibrous tumor
GIST: gastrointestinal stromal tumor
ASPS: alveolar soft part sarcoma
